# Supplementary material for: The Association of Vitamin D and Its Pathway Genes’ Polymorphisms with Hypertensive Disorders of Pregnancy: A Prospective Cohort Study
Source: Nutrients. 2022 Jun 6;14(11):2355. doi: 10.3390/nu14112355 (PMC9183033; doi:10.3390/nu14112355)
Supplement: Supplementary file 1 [file nutrients-14-02355-s001.zip › nutrients-1717678-supplementary.pdf]

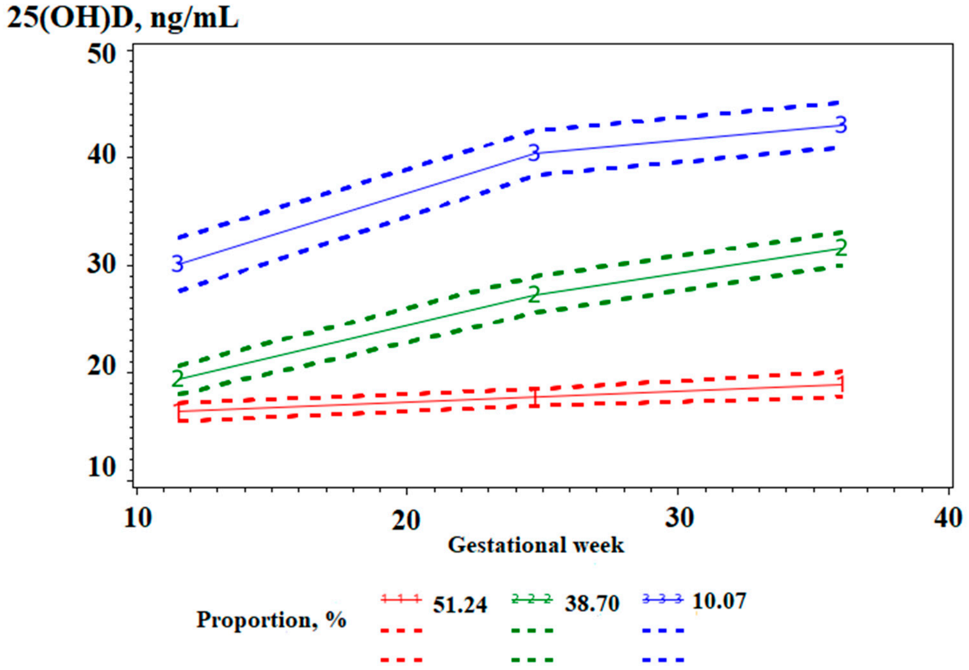

Figure S1. Trajectory of 25(OH)D level during pregnancy

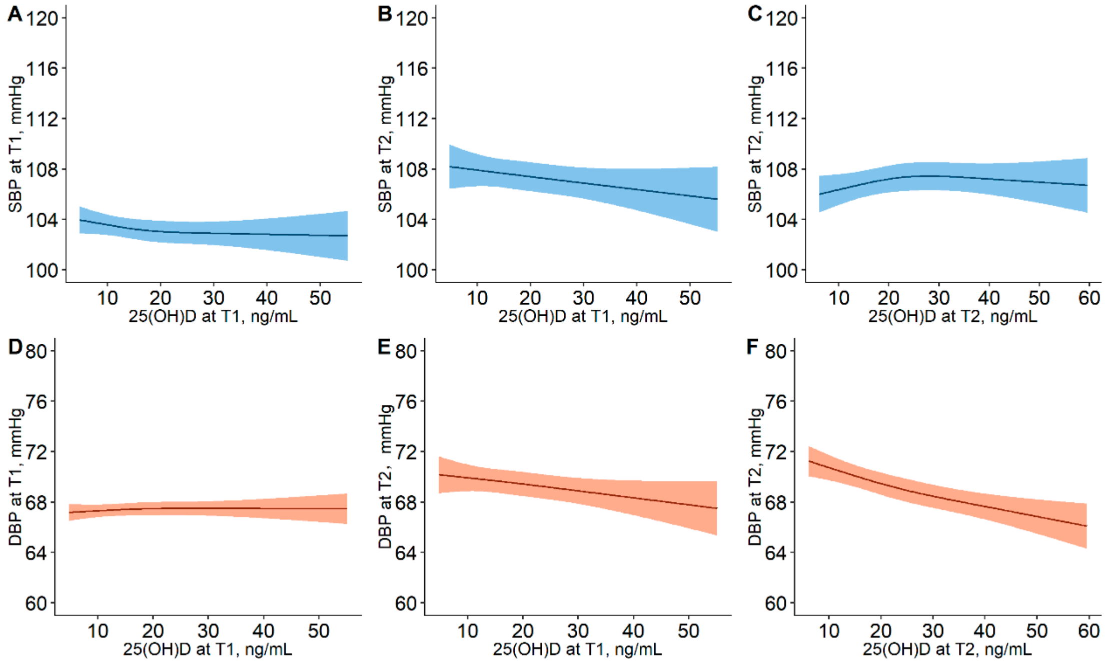

Figure S2. Dose-response relationships of 25(OH)D levels at T1 and T2 with blood pressure at T1 and T2.

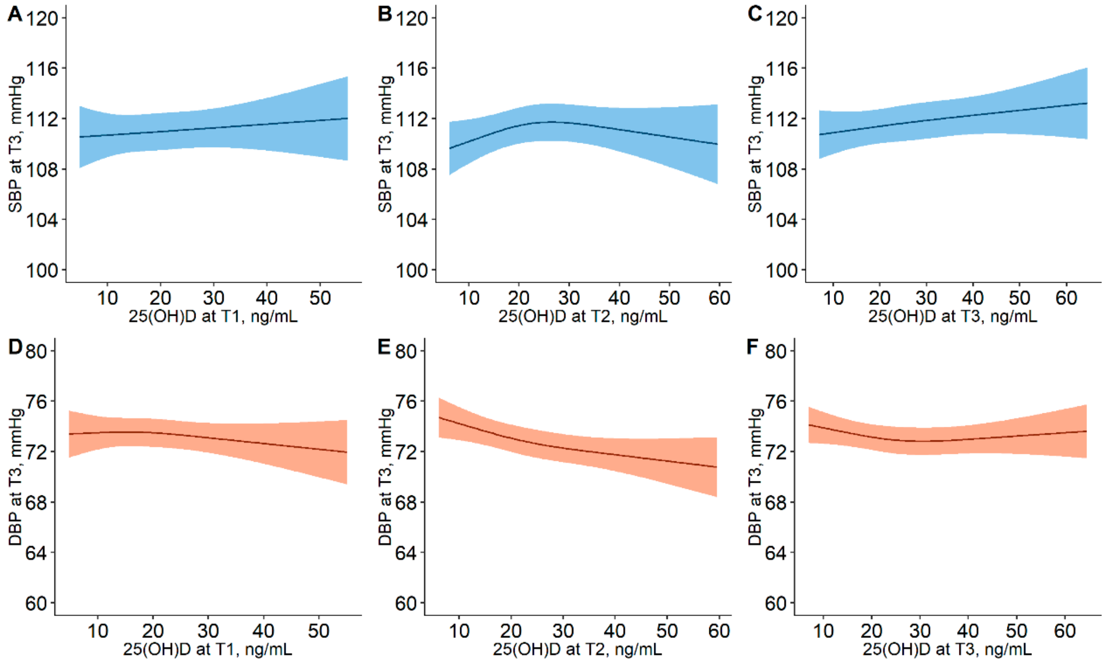

Figure S3. Dose-response relationships of 25(OH)D levels at T1, T2 and T3 with blood pressure at T3.

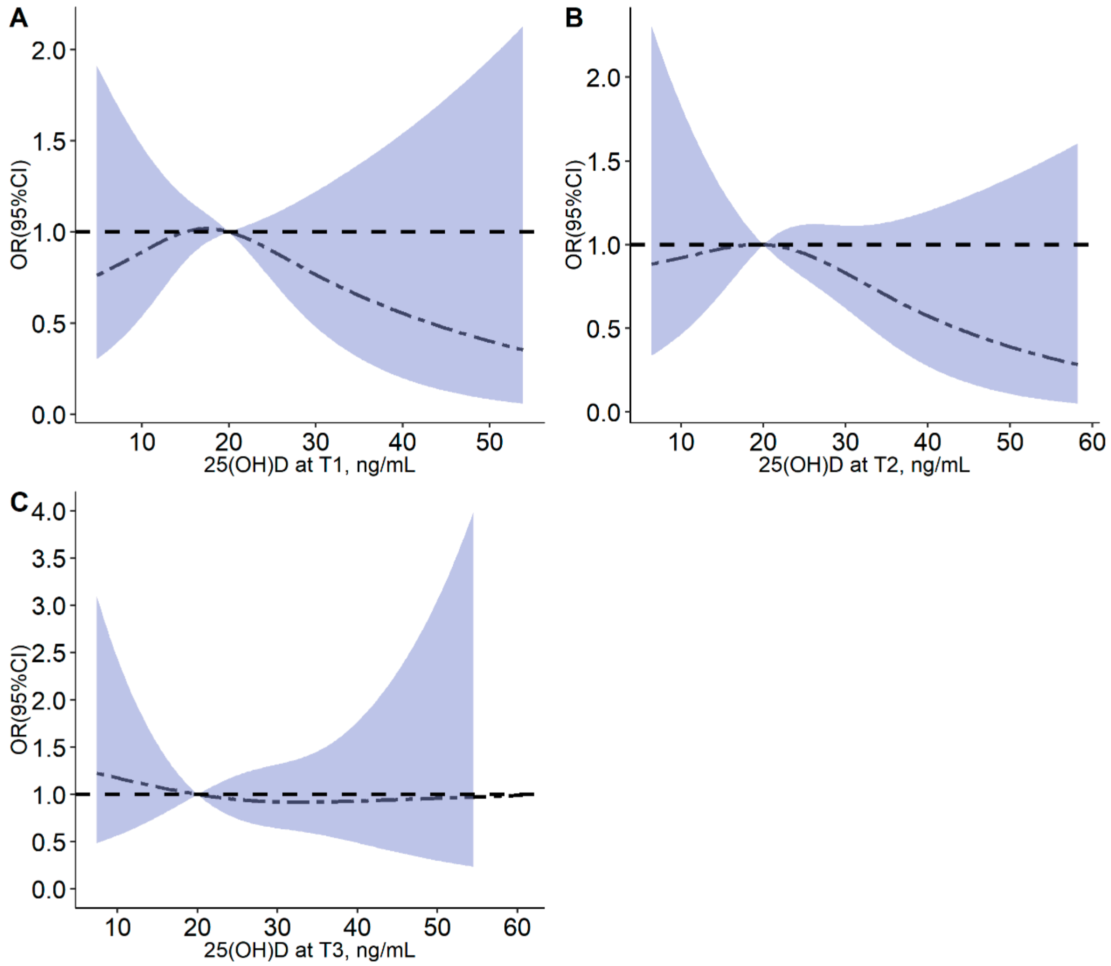

Figure S4. Dose-response relationships of 25(OH)D levels at T1, T2 and T3 with HDP.

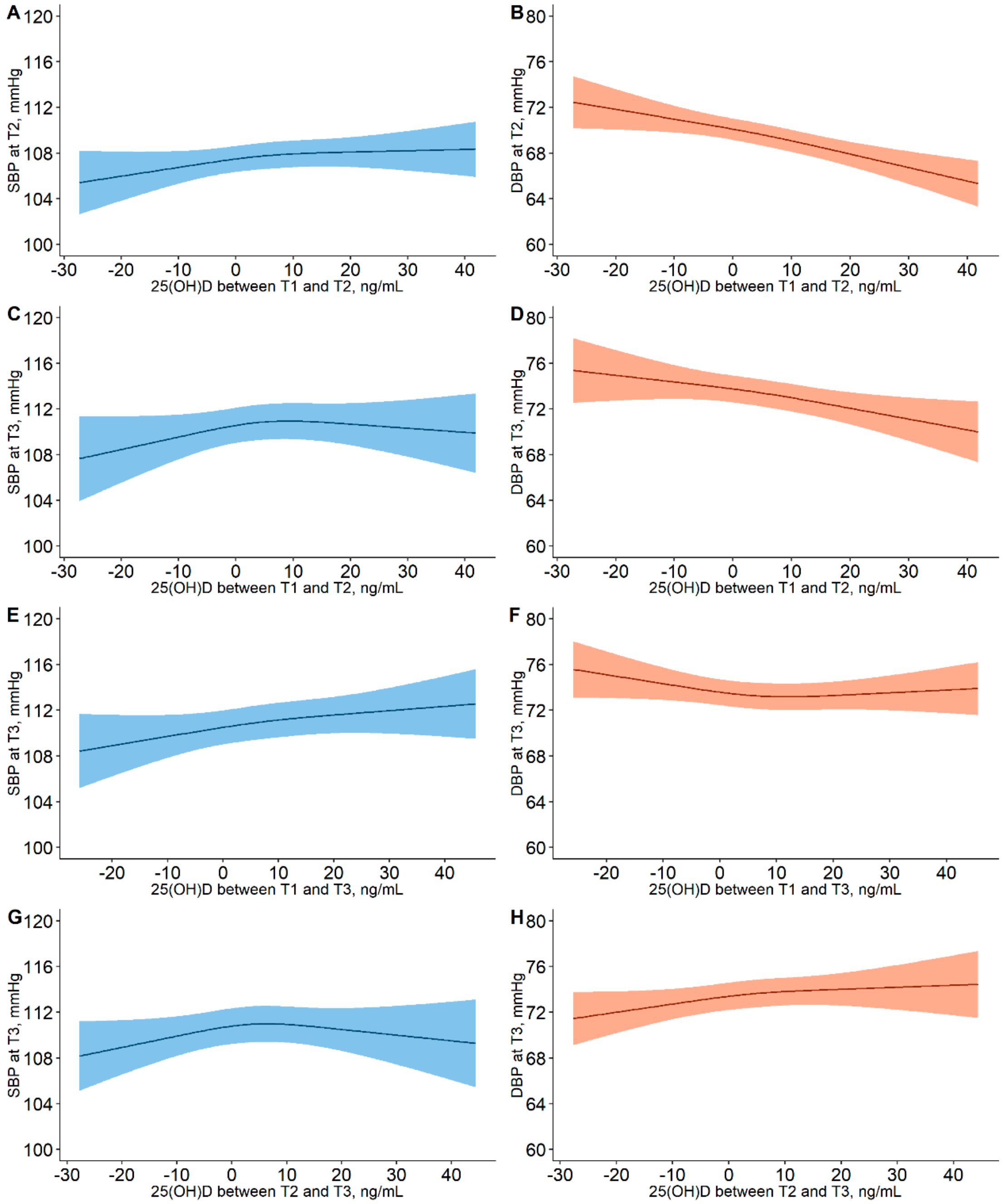

Figure S5. Dose-response relationships of the change of 25(OH)D levels during pregnancy with blood pressure at T2 and T3.

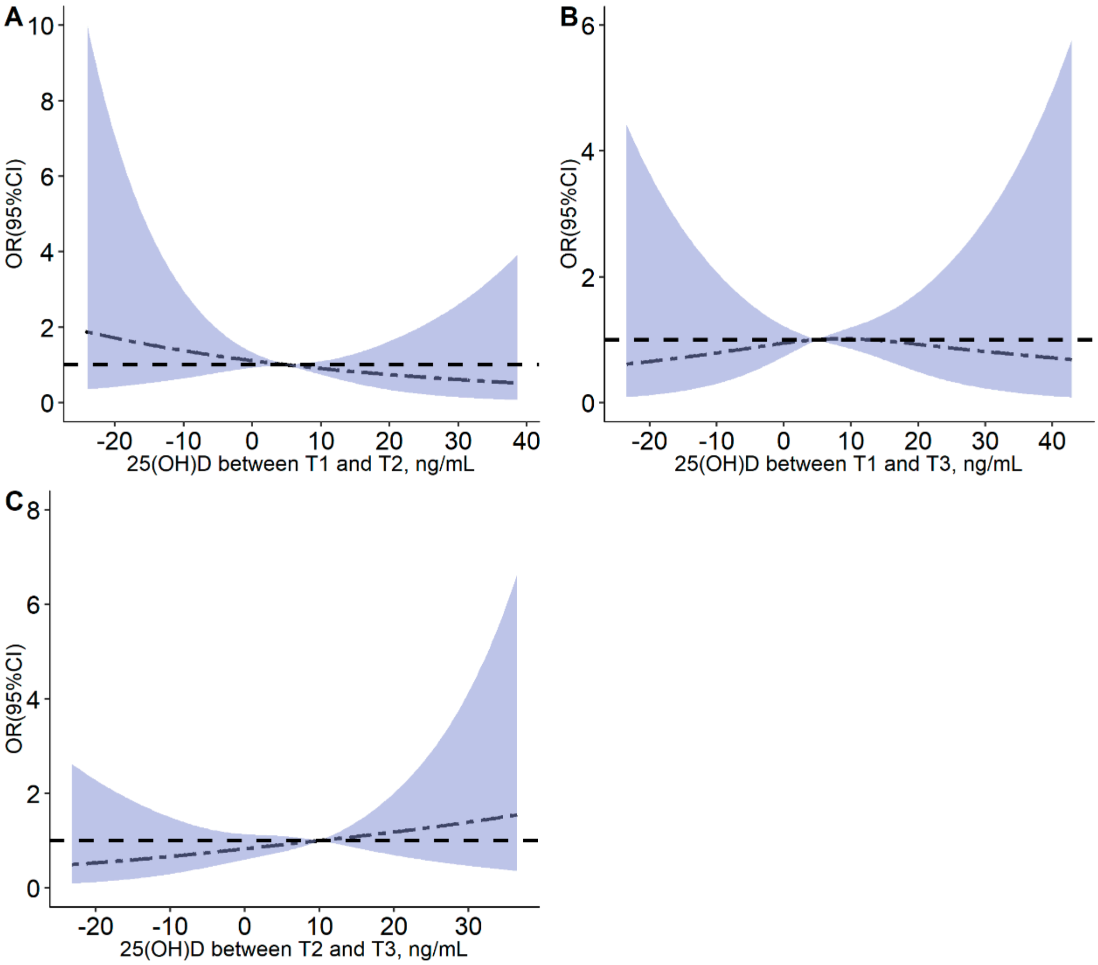

Figure S6. Dose-response relationships between the change of 25(OH)D levels during pregnancy and HDP.

Table S1. Baseline characteristics of pregnant women in SNP analysis.

Variables	Non-HDP (N=2638)	HDP (N=72)	P *
	Mean ± SD		
Age, years	28.73±3.63	29.71±4.11	0.0242
Pre-pregnancy BMI, kg/m^2	21.13±2.91	23.79±4.22	<.0001
T1 (N=2464)			
Weight gain, kg	0.00±0.18	0.01±0.12	0.6163
SBP, mmHg	103.36±9.15	112.87±11.09	<.0001
DBP, mmHg	68.33±6.67	74.27±6.13	<.0001
25(OH)D, ng/mL	18.37±8.57	17.78±7.10	0.5774
T2 (N=1965)			
Weight gain, kg	5.53±3.76	6.14±4.50	0.253
SBP, mmHg	107.27±9.31	116.67±15.00	<.0001
DBP, mmHg	69.18±7.82	76.62±8.97	<.0001
25(OH)D, ng/mL	23.38±10.39	23.55±9.93	0.9093
T3 (N=1263)			
Weight gain, kg	11.84±3.70	11.32±4.19	0.4063
SBP, mmHg	108.84±9.75	121.69±15.52	<.0001
DBP, mmHg	70.80±7.52	81.81±8.18	<.0001
25(OH)D, ng/mL	26.45±11.18	27.33±11.17	0.6422
	N (%)		
VitD deficiency at T1 [a]	1554 (64.83)	46 (68.66)	0.5175
VitD deficiency at T2 [b]	829 (43.34)	24 (46.15)	0.6857
VitD deficiency at T3 [c]	396 (32.27)	10 (27.78)	0.5691
Educational level			0.2972
≤High school	732 (27.75)	24 (33.33)	
>High school	1906 (72.25)	48 (66.67)	
Gravity			0.7966
1	1228 (46.55)	31 (43.06)	
≥2	1324 (50.19)	39 (54.17)	
Unknown	86 (3.26)	2 (2.78)	
Parity			0.7934
0	1442 (54.66)	42 (58.33)	
≥1	591 (22.40)	14 (19.44)	
Unknown	605 (22.93)	16 (22.22)	

Abbreviations: HDP, hypertensive disorders in pregnancy; BMI, body mass index; SBP, systolic blood pressure; DBP, diastolic blood pressure; VitD, vitamin D.

[a] N=2464, [b] N=1965, [c] N=1263

Table S2. The relationship of VitD deficiency in three trimesters with blood pressure.

Variables	SBP, mmHg		DBP, mmHg	
	β(se)	*P*	β(se)	*P*
**SBP at T1（N=3302）**				
VitD deficiency at T1				
No	Ref		Ref	
Yes	0.15 (0.35)	0.6666	-0.20 (0.25)	0.4291
**SBP at T2（N=2479）**				
VitD deficiency at T1				
No	Ref		Ref	
Yes	0.87 (0.43)	0.0415	0.81 (0.35)	0.0201
VitD deficiency at T2				
No	Ref		Ref	
Yes	-0.78 (0.41)	0.0595	1.43 (0.34)	<.0001
**SBP at T3（N=1549）**				
VitD deficiency at T1				
No	Ref		Ref	
Yes	-0.44 (0.56)	0.4320	0.43 (0.43)	0.3116
VitD deficiency at T2				
No	Ref		Ref	
Yes	-0.73 (0.56)	0.1992	1.43 (0.43)	0.0009
VitD deficiency at T3				
No	Ref		Ref	
Yes	-0.85 (0.56)	0.1269	0.07 (0.45)	0.8800

Abbreviations: SBP, systolic blood pressure; DBP, diastolic blood pressure; VitD, vitamin D. Adjusted for pre-pregnancy BMI, maternal age, gestational weight gain, gestational week, educational level, parity, basal blood pressure and the seasons of blood pressure measurement.

Table S3. The association between 25(OH)D levels in three trimesters and HDP.

Trimesters	Non-HDP	HDP	HDP	
	N		OR (95%CI)	*P*
T1	3207	95	1.00 (0.97, 1.03)	0.9083
T2	2417	62	0.99 (0.96, 1.01)	0.2871
T3	1505	44	1.00 (0.97, 1.03)	0.7259

Abbreviations: HDP, hypertensive disorders of pregnancy.

Adjusted for pre-pregnancy BMI, maternal age, gestational weight gain, gestational week, educational level, parity, basal blood pressure and the seasons of blood pressure measurement.

Table S4. The relationship between VitD deficiency in three trimesters with HDP.

Variables	Non-HDP	HDP	HDP	
	N		OR (95%CI)	*P*
VitD deficiency at T1				
No	1031	29	Ref	
Yes	2176	66	1.08 (0.68, 1.72)	0.7479
VitD deficiency at T2				
No	1350	32	Ref	
Yes	1067	30	1.37 (0.80, 2.36)	0.2529
VitD deficiency at T3				
No	1029	31	Ref	
Yes	476	13	1.02 (0.49, 2.11)	0.9616

Abbreviations: HDP, hypertensive disorders of pregnancy; VitD, vitamin D.

Adjusted for pre-pregnancy BMI, maternal age, gestational weight gain, gestational week, educational level, parity, basal blood pressure and the seasons of blood pressure measurement.

Table S5. The association between the change of 25(OH)D levels during pregnancy and HDP.

The change of trimesters	N	the change of 25(OH)D levels, ng/mL *	HDP	
			OR (95%CI)	*P*
From T1 to T2	2125	3.50 (84.59)	0.99 (0.96, 1.02)	0.4123
From T1 to T3	1328	6.59 (98.02)	1.00 (0.97, 1.03)	0.9190
From T2 to T3	1212	3.19 (87.23)	1.02 (0.99, 1.05)	0.2827

Abbreviations: HDP, hypertensive disorders in pregnancy.

* Presented as the median (range).

Adjusted for pre-pregnancy BMI, maternal age, gestational weight gain, gestational week, educational level, parity, basal blood pressure and the seasons of blood pressure measurement.

Table S6. The association between the trajectory of VitD during pregnancy and HDP.

Trajectory of VitD	Non-HDP	HDP	HDP	
	N		OR (95%CI)	*P*
1	602	19	Ref	
2	455	14	0.84 (0.40, 1.75)	0.6418
3	120	2	0.46 (0.10, 2.20)	0.3320

Abbreviations: VitD, vitamin D; HDP, hypertensive disorders in pregnancy.

Adjusted for pre-pregnancy BMI, maternal age, educational level, parity and basal blood pressure.

Table S7. The association of single SNP with SBP and DBP at T1.

SNP	Genotype	N	SBP, mmHg			DBP, mmHg		
			Mean ± SD	β(se)	*P*	Mean ± SD	β(se)	*P*
CYP24A1								
rs2209314	TT	876	103.74±9.46	Ref		68.44±6.69	Ref	
	CT	1181	103.59±9.34	-0.19(0.41)	0.634	68.58±6.75	0.13(0.29)	0.6451
	CC	390	103.52±9.04	-0.27(0.55)	0.6277	68.44±6.76	-0.01(0.40)	0.9812
rs2248137	GG	837	103.57±9.40	Ref		68.86±6.86	Ref	
	GC	434	104.70±10.2	1.34(0.55)	0.015	68.26±6.88	-0.40(0.39)	0.3063
	CC	597	103.14±8.93	-0.24(0.49)	0.6267	68.17±6.61	-0.51(0.35)	0.1524
rs2762934	GG	586	104.33±9.29	Ref		67.90±6.43	Ref	
	GA	117	105.04±11.9	0.55(0.96)	0.5687	66.84±7.36	-1.23(0.64)	0.0565
	AA	7	108.43±12.7	5.45(3.61)	0.1314	71.57±6.65	4.76(2.42)	0.0498
rs6013897	TT	518	104.46±9.41	Ref		67.87±6.55	Ref	
	AT	168	105.23±10.9	0.86(0.84)	0.3092	67.87±6.95	0.02(0.57)	0.9709
	AA	22	100.18±8.14	-3.37(2.07)	0.1032	65.41±4.98	-1.91(1.40)	0.1717
rs6127118	GG	870	103.66±9.26	Ref		68.49±6.74	Ref	

	AG	1466	103.50±9.18	-0.13(0.39)	0.7441	68.57±6.70	0.08(0.28)	0.7666
	AA	116	105.13±11.7	1.33(0.90)	0.1405	67.76±6.87	-0.76(0.65)	0.2384
CYP27B1								
rs10877012	TT	1024	103.74±9.22	Ref		68.76±6.90	Ref	
	GT	1097	103.71±9.73	0.04(0.40)	0.9187	68.36±6.70	-0.32(0.28)	0.265
	GG	322	102.92±8.30	-0.50(0.58)	0.3859	68.20±6.27	-0.34(0.42)	0.4108
CYP3A4								
rs2242480	CC	1389	103.56±9.39	Ref		68.42±6.77	Ref	
	CT	918	103.80±9.15	0.28(0.39)	0.4777	68.60±6.64	0.14(0.28)	0.6161
	TT	141	103.35±10.1	-0.23(0.80)	0.773	68.70±6.67	0.26(0.57)	0.6464
rs4646437	GG	521	104.40±9.65	Ref		67.40±6.23	Ref	
	AG	176	104.93±10.0	0.20(0.83)	0.8058	68.92±7.49	0.74(0.47)	0.1137
	AA	12	102.83±12.8	-0.56(2.78)	0.8398	66.92±7.67	-0.18(1.57)	0.9109
GC								
rs1155563	TT	862	103.75±9.19	Ref		68.52±6.69	Ref	
	TC	1167	103.49±9.28	-0.26(0.41)	0.5237	68.45±6.71	-0.07(0.29)	0.8163
	CC	417	103.84±9.74	0.03(0.54)	0.9594	68.64±6.86	0.03(0.39)	0.9398

rs12512631	TT	456	104.55±9.90	Ref		67.81±6.87	Ref	
	CT	233	104.37±9.73	0.28(0.77)	0.7163	67.75±6.19	0.29(0.52)	0.5697
	CC	18	104.44±8.56	-1.10(2.29)	0.6324	67.50±5.60	-0.78(1.55)	0.6133
rs16846876	AA	1151	103.70±9.53	Ref		68.49±6.76	Ref	
	AT	1038	103.59±9.01	-0.12(0.39)	0.7548	68.46±6.58	-0.02(0.28)	0.9436
	TT	264	103.58±9.78	-0.30(0.62)	0.6281	68.71±7.13	0.05(0.44)	0.9161
rs17467825	AA	1139	103.74±9.44	Ref		68.55±6.77	Ref	
	GA	1043	103.28±9.06	-0.50(0.39)	0.2032	68.26±6.58	-0.32(0.28)	0.2528
	GG	275	104.43±9.86	0.52(0.61)	0.3958	69.18±7.04	0.44(0.44)	0.3126
rs2070741	TT	485	104.74±9.68	Ref		68.05±6.48	Ref	
	GT	209	103.83±10.1	-0.73(0.78)	0.354	67.04±6.87	-0.86(0.53)	0.1037
	GG	17	105.59±9.10	-0.15(2.33)	0.9494	67.82±6.69	-0.91(1.58)	0.5649
rs222020	TT	264	103.98±8.93	Ref		67.55±6.23	Ref	
	CT	339	104.58±10.2	0.53(0.78)	0.4936	68.04±6.98	0.42(0.52)	0.4261
	CC	106	105.54±10.7	1.50(1.09)	0.1691	67.36±6.33	-0.28(0.73)	0.7037
rs2282679	TT	1137	103.76±9.46	Ref		68.54±6.79	Ref	
	GT	1038	103.31±9.05	-0.49(0.39)	0.2096	68.29±6.57	-0.27(0.28)	0.3294

	GG	280	104.35±9.83	0.40(0.61)	0.5052	69.13±7.02	0.39(0.43)	0.3669
rs2298849	AA	1024	103.59±9.30	Ref		68.62±6.72	Ref	
	GA	1111	103.52±9.18	-0.14(0.39)	0.7309	68.48±6.75	-0.20(0.28)	0.4784
	GG	324	104.04±9.92	0.27(0.58)	0.6368	68.17±6.67	-0.60(0.41)	0.1499
rs2298850	GG	1113	103.79±9.49	Ref		68.59±6.81	Ref	
	CG	1048	103.26±9.04	-0.55(0.39)	0.1591	68.25±6.56	-0.35(0.28)	0.217
	CC	278	104.37±9.86	0.41(0.61)	0.5029	69.10±7.02	0.34(0.44)	0.4403
rs3755967	CC	1133	103.77±9.45	Ref		68.55±6.79	Ref	
	CT	1046	103.29±9.04	-0.50(0.39)	0.1989	68.28±6.56	-0.29(0.28)	0.3026
	TT	280	104.35±9.83	0.40(0.61)	0.5088	69.13±7.02	0.38(0.43)	0.3758
rs4588	GG	1124	103.79±9.47	Ref		68.60±6.80	Ref	
	GT	1044	103.28±9.04	-0.52(0.39)	0.1803	68.24±6.57	-0.36(0.28)	0.1972
	TT	279	104.36±9.84	0.39(0.61)	0.5197	69.09±7.01	0.31(0.44)	0.478
rs7041	AA	1315	103.61±9.24	Ref		68.53±6.76	Ref	
	CA	970	103.46±9.28	-0.05(0.38)	0.9036	68.40±6.64	-0.03(0.28)	0.9146
	CC	170	104.57±10.3	1.10(0.74)	0.137	68.76±7.04	0.34(0.53)	0.5244
LRP2								

rs10210408	CC	815	103.48±9.01	Ref		68.43±6.63	Ref	
	TC	1200	103.65±9.39	0.13(0.41)	0.7513	68.60±6.73	0.12(0.30)	0.6733
	TT	445	103.87±9.75	0.35(0.53)	0.5183	68.33±6.88	-0.12(0.38)	0.7632
rs2228171	TT	839	103.38±8.86	Ref		68.52±6.65	Ref	
	CT	378	104.78±10.1	1.56(0.56)	0.0057	67.65±6.70	-0.68(0.40)	0.0877
	CC	278	105.02±9.86	1.80(0.63)	0.0041	68.55±6.62	0.21(0.44)	0.6335
rs2389557	AA	191	103.64±9.28	Ref		67.81±6.51	Ref	
	GA	355	105.02±10.2	1.53(0.85)	0.0718	67.81±6.61	0.13(0.57)	0.8263
	GG	162	104.50±9.34	0.76(1.01)	0.4537	67.69±6.78	-0.16(0.68)	0.8205
rs2544381	GG	382	104.54±9.88	Ref		68.03±6.72	Ref	
	CG	275	104.46±9.81	0.35(0.75)	0.647	67.46±6.57	-0.27(0.51)	0.5979
	CC	52	104.38±9.35	-0.16(1.41)	0.9106	67.37±6.03	-0.64(0.95)	0.4972
rs2544390	CC	195	104.39±9.53	Ref		67.83±6.50	Ref	
	CT	361	104.35±9.66	0.00(0.85)	0.9983	67.87±6.80	0.08(0.57)	0.8833
	TT	152	104.95±10.5	0.65(1.03)	0.5292	67.46±6.29	-0.30(0.69)	0.6654
rs4667591	TT	239	103.13±9.54	Ref		67.33±6.49	Ref	
	GT	339	105.06±9.95	2.18(0.80)	0.0065	67.82±6.66	0.58(0.54)	0.2849

	GG	131	105.54±9.71	2.63(1.03)	0.0106	68.50±6.68	1.21(0.70)	0.082
rs7600336	CC	228	103.50±8.82	Ref		67.38±6.04	Ref	
	TC	338	104.97±9.74	1.51(0.81)	0.0624	68.09±6.80	0.72(0.55)	0.1899
	TT	145	104.92±11.2	1.45(1.00)	0.1489	67.52±7.01	0.15(0.68)	0.8282
VDR								
rs10783219	AA	911	103.65±9.22	Ref		68.39±6.71	Ref	
	TA	1151	103.47±8.99	-0.29(0.40)	0.4765	68.60±6.68	0.12(0.29)	0.6849
	TT	393	104.00±10.5	0.42(0.55)	0.4481	68.44±6.88	0.10(0.39)	0.7918
rs11568820	CC	216	105.33±11.3	Ref		67.94±7.25	Ref	
	TC	341	104.31±9.23	-0.92(0.82)	0.2663	67.93±6.36	0.09(0.55)	0.8753
	TT	150	103.53±8.66	-1.41(1.01)	0.163	67.03±6.13	-0.68(0.68)	0.3164
rs2228570	GG	208	105.00±9.98	Ref		67.80±6.59	Ref	
	GA	357	104.59±9.89	-0.29(0.83)	0.7252	67.82±6.65	0.12(0.56)	0.8289
	AA	144	103.56±9.32	-1.03(1.03)	0.3168	67.55±6.58	0.05(0.69)	0.9374
rs2238136	CC	474	104.58±9.97	Ref		67.76±6.66	Ref	
	TC	212	104.30±9.61	-0.37(0.79)	0.6399	67.40±6.46	-0.36(0.53)	0.4895
	TT	24	104.75±8.13	0.26(1.98)	0.8949	71.33±6.10	3.58(1.33)	0.0073

rs2853559	GG	315	104.64±9.90	Ref		67.91±6.98	Ref	
	GA	303	104.05±9.41	-0.69(0.76)	0.3656	67.65±6.32	-0.33(0.51)	0.5193
	AA	86	105.62±10.8	0.56(1.15)	0.625	67.74±6.32	-0.41(0.78)	0.6029
rs4334089	GG	225	104.88±11.0	Ref		67.58±6.98	Ref	
	AG	340	104.33±9.27	-0.39(0.81)	0.6324	68.10±6.50	0.66(0.55)	0.2269
	AA	145	104.26±9.07	-0.38(1.01)	0.7083	67.23±6.23	-0.20(0.68)	0.7694
rs7975232	CC	371	104.55±9.83	Ref		67.57±6.63	Ref	
	CA	281	103.86±9.85	-0.83(0.75)	0.2636	67.34±6.35	-0.33(0.50)	0.5144
	AA	60	107.05±8.95	2.10(1.32)	0.1117	70.87±6.98	3.00(0.88)	0.0007

Abbreviations: VitD, vitamin D; SBP, systolic blood pressure; DBP, diastolic blood pressure.

Adjusted for pre-pregnancy BMI, maternal age, gestational weight gain, educational level, parity, basal blood pressure and the seasons of blood pressure measurement.

Table S8. The association of single SNP with SBP and DBP at T2.

SNP	Genotypes	N	SBP, mmHg			DBP, mmHg		
			Mean ± SD	β(se)	*P*	Mean ± SD	β(se)	*P*
CYP24A1								
rs2209314	TT	692	107.16±9.50	Ref		69.74±8.06	Ref	
	CT	941	107.80±9.94	0.68(0.47)	0.1473	69.40±7.91	-0.35(0.39)	0.367
	CC	324	107.39±8.88	0.24(0.63)	0.7061	68.40±7.65	-1.31(0.52)	0.0116
rs2248137	GG	734	107.90±10.1	Ref		68.25±8.04	Ref	
	GC	244	106.87±9.00	-1.16(0.73)	0.1124	71.19±6.97	2.17(0.59)	0.0003
	CC	440	107.25±9.24	-0.41(0.57)	0.477	68.96±7.91	0.73(0.46)	0.1133
rs2762934	GG	269	106.03±7.54	Ref		71.97±6.15	Ref	
	GA	57	108.04±9.23	2.26(1.12)	0.0442	72.56±6.59	0.75(0.90)	0.4055
	AA	3	107.33±6.43	2.20(4.47)	0.6227	76.67±5.77	4.89(3.58)	0.1728
rs6013897	TT	251	106.65±7.85	Ref		72.02±6.35	Ref	
	AT	62	106.44±7.54	-0.07(1.10)	0.9474	72.61±5.64	0.72(0.88)	0.4168
	AA	13	102.62±9.61	-3.30(2.22)	0.1373	72.15±7.50	0.74(1.77)	0.6771
rs6127118	GG	696	107.73±9.17	Ref		69.14±7.68	Ref	

	AG	1207	107.43±9.94	-0.40(0.45)	0.3667	69.35±8.13	0.22(0.37)	0.5413
	AA	53	106.98±8.32	-1.40(1.35)	0.3008	73.25±5.58	2.65(1.11)	0.0167
CYP27B1								
rs10877012	TT	806	107.45±9.60	Ref		69.14±8.10	Ref	
	GT	880	107.60±9.56	0.22(0.46)	0.6313	69.65±7.62	0.48(0.38)	0.201
	GG	265	107.38±9.97	0.06(0.67)	0.9307	69.19±8.41	0.18(0.55)	0.7431
CYP3A4								
rs2242480	CC	1124	107.69±9.86	Ref		69.61±8.00	Ref	
	CT	722	107.15±9.24	-0.32(0.45)	0.4775	69.03±7.89	-0.43(0.37)	0.2384
	TT	110	107.88±9.29	0.30(0.94)	0.7483	68.99±7.45	-0.86(0.77)	0.2623
rs4646437	GG	243	106.33±7.99	Ref		72.33±6.29	Ref	
	AG	80	106.90±7.61	0.40(1.00)	0.6886	71.88±5.99	-0.50(0.79)	0.5272
	AA	4	100.00±0.00	-5.92(3.90)	0.13	65.00±5.77	-7.50(3.10)	0.0161
GC								
rs1155563	TT	712	107.66±9.64	Ref		69.42±7.85	Ref	
	TC	901	107.46±9.54	-0.30(0.47)	0.529	69.37±7.98	-0.19(0.39)	0.6327
	CC	339	107.25±9.81	-0.51(0.62)	0.4074	69.28±8.04	-0.18(0.51)	0.7224

rs12512631	TT	212	106.44±8.24	Ref		72.11±6.02	Ref	
	CT	105	106.10±7.25	-0.02(0.94)	0.981	71.89±6.79	-0.02(0.75)	0.974
	CC	8	108.00±5.13	-0.15(2.81)	0.9566	75.00±4.41	1.87(2.25)	0.4058
rs16846876	AA	936	107.71±9.51	Ref		69.51±7.89	Ref	
	AT	812	107.24±9.63	-0.51(0.45)	0.2548	69.05±7.99	-0.45(0.37)	0.2191
	TT	210	107.77±10.1	-0.17(0.72)	0.8084	70.02±8.00	0.51(0.59)	0.3899
rs17467825	AA	925	107.83±9.57	Ref		69.43±7.91	Ref	
	GA	820	107.10±9.44	-0.82(0.45)	0.0667	69.19±7.91	-0.26(0.37)	0.4876
	GG	215	107.74±10.4	-0.38(0.71)	0.5973	69.76±8.15	0.27(0.58)	0.6503
rs2070741	TT	218	105.54±7.46	Ref		72.00±6.20	Ref	
	GT	101	107.77±8.22	2.13(0.95)	0.0256	72.22±6.26	0.36(0.77)	0.639
	GG	10	111.00±9.94	5.10(2.48)	0.0405	73.50±6.92	0.92(2.00)	0.6475
rs222020	TT	127	105.05±7.99	Ref		71.93±6.27	Ref	
	CT	147	106.67±7.95	1.29(0.93)	0.167	72.47±6.25	0.59(0.75)	0.4338
	CC	54	108.67±6.83	3.65(1.25)	0.0037	71.63±6.19	-0.01(1.01)	0.9921
rs2282679	TT	923	107.84±9.57	Ref		69.42±7.94	Ref	
	GT	815	107.14±9.48	-0.78(0.45)	0.0818	69.25±7.89	-0.21(0.37)	0.5632

	GG	221	107.63±10.3	-0.48(0.70)	0.496	69.67±8.14	0.19(0.58)	0.7393
rs2298849	AA	808	106.96±9.82	Ref		69.18±7.96	Ref	
	GA	873	108.00±9.62	1.01(0.46)	0.0271	69.43±7.98	0.24(0.38)	0.5158
	GG	281	107.60±8.91	0.74(0.65)	0.256	69.79±7.79	0.66(0.53)	0.2138
rs2298850	GG	903	107.82±9.60	Ref		69.49±7.88	Ref	
	CG	822	107.12±9.45	-0.77(0.45)	0.0894	69.24±7.90	-0.27(0.37)	0.4731
	CC	221	107.61±10.4	-0.47(0.70)	0.509	69.67±8.14	0.13(0.58)	0.8162
rs3755967	CC	920	107.83±9.57	Ref		69.43±7.94	Ref	
	CT	821	107.14±9.46	-0.77(0.45)	0.0858	69.23±7.88	-0.22(0.37)	0.5439
	TT	221	107.63±10.3	-0.48(0.70)	0.4982	69.67±8.14	0.19(0.58)	0.7376
rs4588	GG	912	107.82±9.57	Ref		69.42±7.93	Ref	
	GT	821	107.14±9.46	-0.74(0.45)	0.0988	69.27±7.91	-0.18(0.37)	0.6232
	TT	222	107.59±10.3	-0.50(0.70)	0.4725	69.58±8.12	0.10(0.58)	0.8584
rs7041	AA	1051	107.78±9.91	Ref		69.54±7.83	Ref	
	CA	769	107.26±9.36	-0.32(0.45)	0.4781	69.27±8.08	-0.25(0.37)	0.4904
	CC	140	106.66±8.65	-0.92(0.84)	0.2736	68.56±7.84	-0.84(0.69)	0.2233
LRP2								

rs10210408	CC	648	107.58±9.33	Ref		69.48±7.51	Ref	
	TC	957	107.37±9.81	-0.21(0.48)	0.6529	69.26±8.14	-0.16(0.39)	0.6752
	TT	357	107.85±9.62	0.27(0.62)	0.6657	69.47±8.17	-0.09(0.51)	0.8639
rs2228171	TT	668	107.17±9.30	Ref		69.41±7.62	Ref	
	CT	185	106.23±8.83	-0.96(0.75)	0.2012	71.61±6.63	1.43(0.62)	0.0212
	CC	215	106.80±9.09	-0.27(0.70)	0.7044	70.73±7.90	0.89(0.58)	0.125
rs2389557	AA	93	106.75±7.16	Ref		71.43±6.28	Ref	
	GA	165	106.05±8.41	-0.50(1.01)	0.6208	72.37±6.39	0.86(0.80)	0.2835
	GG	68	106.81±7.54	0.20(1.24)	0.8701	72.53±5.87	1.01(0.99)	0.3098
rs2544381	GG	184	106.46±8.18	Ref		72.03±6.32	Ref	
	CG	121	106.40±7.65	0.38(0.91)	0.6798	71.89±5.96	-0.05(0.73)	0.94
	CC	23	105.48±6.67	-1.42(1.72)	0.4103	74.09±6.89	1.51(1.37)	0.2728
rs2544390	CC	97	106.76±7.44	Ref		71.57±5.95	Ref	
	CT	159	106.18±8.17	-0.32(1.00)	0.7521	72.25±6.25	0.74(0.80)	0.3523
	TT	71	106.37±7.88	-0.12(1.21)	0.9194	72.63±6.64	0.99(0.97)	0.3059
rs4667591	TT	99	106.34±7.25	Ref		72.23±6.31	Ref	
	GT	163	105.84±8.12	-0.50(1.00)	0.6172	71.71±6.42	-0.61(0.80)	0.4467

	GG	65	107.85±8.10	1.51(1.26)	0.2312	73.02±5.66	1.00(1.01)	0.3222
rs7600336	CC	106	105.68±6.97	Ref		71.92±6.21	Ref	
	TC	149	106.64±8.41	0.96(1.00)	0.3355	72.29±6.41	0.51(0.80)	0.5232
	TT	74	106.89±7.98	1.32(1.18)	0.2652	72.05±5.97	0.36(0.94)	0.7005
VDR								
rs10783219	AA	728	107.44±9.70	Ref		69.38±7.83	Ref	
	TA	942	107.92±9.66	0.33(0.46)	0.4695	69.27±8.02	-0.14(0.38)	0.713
	TT	291	106.43±9.24	-0.91(0.65)	0.1623	69.75±7.91	0.40(0.54)	0.4541
rs11568820	CC	84	105.89±7.03	Ref		71.81±6.36	Ref	
	TC	168	105.92±8.24	0.15(1.04)	0.8836	72.24±6.42	0.54(0.83)	0.5187
	TT	74	107.88±7.76	1.59(1.24)	0.2003	72.12±5.73	0.33(0.99)	0.7406
rs2228570	GG	88	107.20±8.44	Ref		72.36±6.01	Ref	
	GA	180	106.13±7.78	-1.17(1.01)	0.2457	72.12±6.35	-0.47(0.81)	0.5616
	AA	60	105.87±7.31	-0.72(1.31)	0.5796	71.77±6.30	-0.59(1.04)	0.5754
rs2238136	CC	225	106.36±8.03	Ref		72.23±6.14	Ref	
	TC	94	106.27±7.37	0.44(0.97)	0.6516	71.56±6.40	-0.34(0.77)	0.6588
	TT	9	107.78±9.72	1.08(2.65)	0.6835	75.33±6.48	2.46(2.11)	0.2451

rs2853559	GG	145	106.19±8.16	Ref		72.49±6.58	Ref	
	GA	142	106.60±7.84	0.26(0.92)	0.774	71.93±6.14	-0.55(0.73)	0.4562
	AA	37	106.11±6.91	-0.73(1.44)	0.6138	71.11±5.21	-1.46(1.15)	0.2063
rs4334089	GG	93	105.57±7.13	Ref		71.51±6.64	Ref	—
	AG	162	106.19±8.24	1.00(1.02)	0.3239	72.33±6.45	1.00(0.81)	0.2212
	AA	74	107.85±7.82	1.87(1.21)	0.1232	72.42±5.15	0.91(0.97)	0.3464
rs7975232	CC	167	106.71±7.50	Ref		72.57±6.22	Ref	
	CA	129	105.94±8.31	-0.97(0.91)	0.2883	71.31±5.99	-1.28(0.72)	0.0756
	AA	33	106.55±8.05	-0.54(1.48)	0.7138	72.97±7.00	0.50(1.17)	0.668

Abbreviations: VitD, vitamin D; SBP, systolic blood pressure; DBP, diastolic blood pressure.

Adjusted for pre-pregnancy BMI, maternal age, gestational weight gain, educational level, parity, basal blood pressure and the seasons of blood pressure measurement.

Table S9. The association of single SNP with SBP and DBP at T3.

SNP	Genotypes	N	SBP, mmHg			DBP, mmHg		
			Mean ± SD	β(se)	*P*	Mean ± SD	β(se)	*P*
CYP24A1								
rs2209314	TT	439	109.39±10.0	Ref		71.36±8.01	Ref	
	CT	614	109.32±10.5	-0.03(0.62)	0.965	71.09±7.59	-0.31(0.47)	0.5074
	CC	203	108.55±9.63	-0.48(0.84)	0.5669	70.73±7.78	-0.43(0.64)	0.5027
rs2248137	GG	470	109.72±10.6	Ref		70.38±7.93	Ref	
	GC	169	108.74±9.11	-1.00(0.90)	0.2676	72.78±6.56	1.71(0.70)	0.0141
	CC	285	108.71±9.94	-1.05(0.74)	0.1561	70.51±8.18	0.04(0.57)	0.9436
rs2762934	GG	185	107.76±8.69	Ref		72.68±6.28	Ref	
	GA	43	106.33±7.94	-1.36(1.44)	0.3484	71.44±5.91	-1.05(1.06)	0.3226
	AA	4	113.50±4.73	5.89(4.38)	0.1796	76.00±7.12	4.18(3.20)	0.1926
rs6013897	TT	171	107.62±8.47	Ref		72.74±6.16	Ref	
	AT	50	107.76±9.22	0.59(1.42)	0.6761	71.84±6.50	-1.15(1.03)	0.2638
	AA	11	108.18±7.51	0.15(2.72)	0.9575	71.64±6.19	-0.98(1.97)	0.6192
rs6127118	GG	449	108.93±9.54	Ref		71.11±7.67	Ref	

	AG	760	109.54±10.6	0.29(0.59)	0.6217	71.04±7.90	-0.20(0.45)	0.6556
	AA	48	107.08±9.02	-2.36(1.52)	0.1199	72.56±6.11	0.53(1.15)	0.6482
CYP27B1								
rs10877012	TT	499	109.38±10.1	Ref		71.20±7.94	Ref	
	GT	585	109.06±10.3	-0.41(0.61)	0.5019	70.79±7.70	-0.52(0.46)	0.2562
	GG	169	109.03±10.3	-0.30(0.89)	0.7382	71.97±7.33	0.82(0.67)	0.2192
CYP3A4								
rs2242480	CC	751	109.58±10.3	Ref		71.22±7.81	Ref	
	CT	435	108.66±9.78	-0.76(0.60)	0.2068	70.81±7.63	-0.15(0.46)	0.7379
	TT	69	108.87±11.3	-0.75(1.25)	0.5477	72.07±7.95	0.63(0.95)	0.5055
rs4646437	GG	176	107.32±8.77	Ref		72.40±6.49	Ref	
	AG	54	108.74±7.97	1.20(1.34)	0.3706	72.87±5.39	0.38(0.98)	0.6944
	AA	2	105.00±7.07	-1.47(6.11)	0.8106	75.00±7.07	1.95(4.45)	0.6616
GC								
rs1155563	TT	449	109.39±10.4	Ref		71.42±7.74	Ref	
	TC	591	108.80±10.1	-0.50(0.62)	0.4255	70.83±7.78	-0.63(0.47)	0.1863
	CC	216	109.94±10.1	0.57(0.82)	0.4909	71.34±7.73	0.02(0.63)	0.9691

rs12512631	TT	143	108.70±8.68	Ref		72.69±6.12	Ref	
	CT	80	105.59±8.17	-3.11(1.18)	0.0091	72.16±6.61	-0.46(0.87)	0.5988
	CC	8	107.50±7.07	-1.85(3.12)	0.5532	72.50±4.63	-1.03(2.31)	0.6545
rs16846876	AA	587	109.45±10.6	Ref		71.40±7.85	Ref	
	AT	535	108.55±9.49	-1.04(0.60)	0.0806	70.59±7.72	-0.86(0.45)	0.0588
	TT	136	110.94±10.8	1.11(0.95)	0.2417	72.04±7.38	0.71(0.72)	0.3212
rs17467825	AA	582	109.56±10.5	Ref		71.19±7.83	Ref	
	GA	528	108.42±9.66	-1.31(0.60)	0.0292	70.65±7.70	-0.61(0.45)	0.1788
	GG	150	110.53±10.6	0.61(0.91)	0.4987	72.45±7.53	1.13(0.69)	0.1008
rs2070741	TT	150	107.25±8.31	Ref		72.89±6.29	Ref	
	GT	74	108.16±9.06	1.67(1.23)	0.1774	71.62±6.23	-0.73(0.90)	0.4184
	GG	8	108.75±8.35	2.42(3.13)	0.44	73.50±4.87	1.00(2.29)	0.6625
rs222020	TT	79	107.97±8.85	Ref		73.24±6.44	Ref	
	CT	105	107.73±8.17	0.13(1.29)	0.9188	72.42±5.95	-0.59(0.94)	0.5308
	CC	47	106.57±8.98	-0.99(1.63)	0.544	71.53±6.53	-1.34(1.19)	0.2614
rs2282679	TT	582	109.58±10.5	Ref		71.17±7.81	Ref	
	GT	524	108.42±9.65	-1.28(0.60)	0.0325	70.67±7.71	-0.56(0.45)	0.2214

	GG	152	110.61±10.7	0.71(0.90)	0.4296	72.52±7.55	1.27(0.69)	0.065
rs2298849	AA	519	109.79±10.5	Ref		71.29±7.80	Ref	
	GA	564	108.79±9.86	-0.93(0.60)	0.1219	70.85±7.82	-0.49(0.46)	0.2907
	GG	178	108.76±10.4	-0.87(0.87)	0.3156	71.41±7.42	0.19(0.66)	0.7743
rs2298850	GG	566	109.62±10.5	Ref		71.23±7.74	Ref	
	CG	532	108.36±9.63	-1.39(0.60)	0.0208	70.60±7.82	-0.69(0.46)	0.1328
	CC	152	110.61±10.7	0.68(0.90)	0.4526	72.52±7.55	1.21(0.69)	0.0794
rs3755967	CC	580	109.63±10.5	Ref		71.17±7.82	Ref	
	CT	529	108.36±9.63	-1.41(0.60)	0.019	70.65±7.69	-0.58(0.45)	0.2002
	TT	152	110.61±10.7	0.66(0.90)	0.4667	72.52±7.55	1.26(0.69)	0.0653
rs4588	GG	571	109.64±10.5	Ref		71.24±7.74	Ref	
	GT	531	108.39±9.61	-1.37(0.60)	0.0229	70.63±7.78	-0.64(0.46)	0.1593
	TT	153	110.65±10.6	0.67(0.90)	0.455	72.39±7.69	1.07(0.69)	0.1192
rs7041	AA	680	109.33±10.1	Ref		70.87±7.78	Ref	
	CA	495	108.94±10.4	-0.23(0.59)	0.6973	71.35±7.84	0.45(0.45)	0.3092
	CC	84	110.19±9.52	1.13(1.15)	0.3252	71.90±7.03	1.13(0.87)	0.1943
LRP2								

rs10210408	CC	414	109.29±10.3	Ref		71.26±7.58	Ref	
	TC	629	109.03±10.0	-0.35(0.63)	0.5806	70.79±7.84	-0.33(0.48)	0.4964
	TT	218	109.60±10.4	0.40(0.83)	0.6336	71.77±7.81	0.53(0.63)	0.4028
rs2228171	TT	432	109.55±10.5	Ref		71.03±7.72	Ref	
	CT	123	108.11±8.51	-1.68(1.05)	0.1103	72.10±6.77	0.55(0.76)	0.4685
	CC	133	108.11±10.7	-1.17(1.01)	0.249	72.25±7.40	1.15(0.74)	0.1192
rs2389557	AA	73	107.48±8.39	Ref		72.37±6.58	Ref	
	GA	114	107.43±8.52	0.19(1.30)	0.8843	72.39±5.98	0.35(0.94)	0.7113
	GG	44	108.59±9.14	1.01(1.64)	0.5379	73.00±6.39	0.57(1.19)	0.6308
rs2544381	GG	140	107.02±8.08	Ref		72.42±6.48	Ref	
	CG	75	108.99±9.29	2.42(1.23)	0.0501	72.84±6.06	0.72(0.91)	0.4249
	CC	16	105.88±8.66	-0.27(2.28)	0.905	71.88±5.14	-0.55(1.68)	0.7416
rs2544390	CC	75	106.31±8.25	Ref		71.32±6.62	Ref	
	CT	107	108.69±8.50	2.47(1.28)	0.0551	72.85±6.08	1.52(0.93)	0.1041
	TT	49	107.10±8.95	1.27(1.57)	0.4186	73.63±5.81	2.62(1.14)	0.0227
rs4667591	TT	70	108.49±8.32	Ref		71.77±5.06	Ref	
	GT	117	107.68±8.80	-0.85(1.33)	0.5259	72.39±6.63	0.60(0.96)	0.5339

	GG	45	106.18±8.35	-1.70(1.66)	0.3067	74.09±6.70	2.40(1.20)	0.0472
rs7600336	CC	81	107.46±8.52	Ref		71.62±5.90	Ref	
	TC	94	108.59±8.64	0.58(1.31)	0.6591	73.22±6.47	1.48(0.96)	0.1241
	TT	57	106.14±8.31	-1.60(1.47)	0.2801	72.60±6.26	1.08(1.08)	0.3184
VDR								
rs10783219	AA	481	109.19±10.4	Ref		70.93±8.02	Ref	
	TA	591	109.19±10.2	0.04(0.61)	0.9509	71.32±7.81	0.41(0.46)	0.3814
	TT	185	109.37±9.78	0.32(0.86)	0.7104	71.02±6.91	0.16(0.65)	0.8057
rs11568820	CC	56	108.04±9.42	Ref		72.64±5.52	Ref	
	TC	123	107.92±8.19	-0.32(1.37)	0.8146	72.58±6.21	0.11(1.01)	0.9168
	TT	51	105.82±8.07	-2.56(1.64)	0.1214	72.10±7.10	-0.96(1.21)	0.4307
rs2228570	GG	57	107.53±8.15	Ref		72.11±6.33	Ref	
	GA	130	107.20±8.20	-0.51(1.38)	0.7099	73.08±6.39	1.43(1.00)	0.1547
	AA	44	108.77±10.0	1.10(1.76)	0.5347	71.41±5.60	-0.66(1.28)	0.6066
rs2238136	CC	160	107.63±8.71	Ref		72.74±6.49	Ref	
	TC	64	107.25±8.15	-0.33(1.27)	0.7937	71.94±5.77	-0.66(0.92)	0.4765
	TT	8	110.75±9.50	3.49(3.13)	0.2664	73.25±4.65	0.17(2.28)	0.9405

rs2853559	GG	113	107.66±8.47	Ref		72.48±6.61	Ref	
	GA	92	107.18±9.00	-0.41(1.22)	0.7386	72.54±6.21	-0.20(0.89)	0.8211
	AA	24	108.08±7.31	-0.13(1.93)	0.946	72.42±4.72	-0.19(1.41)	0.8945
rs4334089	GG	63	108.13±9.28	Ref		72.43±5.51	Ref	
	AG	120	107.68±7.84	-0.81(1.34)	0.5476	72.57±6.15	0.20(0.98)	0.8391
	AA	49	106.67±9.25	-2.32(1.64)	0.1592	72.47±7.35	-0.57(1.20)	0.6355
rs7975232	CC	123	107.65±8.66	Ref		72.52±6.05	Ref	
	CA	87	107.31±8.41	-0.79(1.21)	0.5111	72.05±6.55	-0.70(0.88)	0.4256
	AA	23	108.87±8.76	0.98(1.98)	0.6223	74.35±5.80	1.58(1.44)	0.2725

Abbreviations: VitD, vitamin D; SBP, systolic blood pressure; DBP, diastolic blood pressure.

Adjusted for pre-pregnancy BMI, maternal age, gestational weight gain, educational level, parity, basal blood pressure and the seasons of blood pressure measurement.

Table S10. The association of single SNP and VitD at T1 with blood pressure at T1.

SNP	Genotypes	VitD deficiency	N	SBP, mmHg			DBP, mmHg		
				Mean ± SD	β(se)	*P*	Mean ± SD	β(se)	*P*
CYP24A1									
rs2762934	GG	No	125	104.44±8.93	Ref		66.95±5.67	Ref	
	GA	No	23	103.91±8.97	-0.64(1.95)	0.7447	67.70±6.60	0.72(1.45)	0.6178
	AA	No	3	115.00±18.0	7.58(5.01)	0.1306	74.33±9.29	8.47(3.71)	0.0227
	GG	Yes	461	104.30±9.39	-1.27(0.88)	0.1497	68.15±6.60	1.02(0.65)	0.1175
	GA	Yes	94	105.32±12.6	-0.56(1.19)	0.6359	66.63±7.55	-0.70(0.88)	0.4270
	AA	Yes	4	103.50±5.51	0.12(4.36)	0.9787	69.50±4.20	3.35(3.23)	0.2995
	P 交互项					0.8672			0.0150

Abbreviations: VitD, vitamin D; SBP, systolic blood pressure; DBP, diastolic blood pressure.

Adjusted for pre-pregnancy BMI, maternal age, gestational weight gain, educational level, parity, basal blood pressure and the seasons of blood pressure measurement.

Table S11. The association of single SNP and VitD at T2 with blood pressure at T2.

SNP	Genotypes	VitD deficiency	N	SBP, mmHg			DBP, mmHg		
				Mean ± SD	β(se)	*P*	Mean ± SD	β(se)	*P*
GC									
rs16846876	AA	No	557	107.92±9.77	Ref		69.34±8.05	Ref	
	AT	No	450	107.41±9.64	-0.62(0.60)	0.2954	68.41±8.25	-0.93(0.49)	0.0565
	TT	No	102	107.61±10.6	-0.66(1.01)	0.5140	68.18±8.23	-1.14(0.83)	0.1703
	AA	Yes	379	107.42±9.13	-0.74(0.63)	0.2397	69.75±7.64	0.25(0.52)	0.6256
	AT	Yes	362	107.04±9.63	-1.05(0.64)	0.1000	69.83±7.58	0.37(0.52)	0.4832
	TT	Yes	108	107.93±9.64	-0.29(0.99)	0.7685	71.76±7.39	2.23(0.81)	0.0059
	$P_{交互项}$					0.4180			0.0056
rs17467825	AA	No	578	107.96±9.83	Ref		69.25±8.00	Ref	
	GA	No	437	107.32±9.58	-0.60(0.59)	0.3020	68.45±8.28	-0.77(0.49)	0.1124
	GG	No	94	107.65±10.5	-0.49(1.03)	0.6368	68.21±8.44	-1.21(0.86)	0.1566
	AA	Yes	347	107.61±9.13	-0.29(0.63)	0.6496	69.71±7.77	0.33(0.53)	0.5284
	GA	Yes	383	106.85±9.28	-1.15(0.61)	0.0613	70.04±7.39	0.60(0.51)	0.2434
	GG	Yes	121	107.81±10.4	-0.36(0.93)	0.6979	70.97±7.74	1.63(0.77)	0.0351

	P 交互项					0.8104			0.0189
rs2282679	TT	No	578	107.95±9.80	Ref		69.26±8.02	Ref	
	GT	No	434	107.37±9.64	-0.55(0.59)	0.3526	68.49±8.27	-0.76(0.49)	0.1180
	GG	No	98	107.40±10.4	-0.71(1.01)	0.4838	68.14±8.35	-1.28(0.84)	0.1291
	TT	Yes	345	107.65±9.17	-0.24(0.63)	0.7038	69.70±7.80	0.29(0.53)	0.5818
	GT	Yes	381	106.87±9.30	-1.11(0.62)	0.0710	70.11±7.34	0.64(0.51)	0.2081
	GG	Yes	123	107.81±10.3	-0.30(0.92)	0.7411	70.88±7.80	1.55(0.77)	0.0429
	P 交互项					0.7543			0.0161
rs2298849	AA	No	430	107.10±10.1	Ref		68.40±8.38	Ref	
	GA	No	511	108.20±9.76	1.17(0.61)	0.0575	69.04±7.95	0.67(0.50)	0.1752
	GG	No	170	107.59±8.99	0.59(0.85)	0.4894	69.59±8.24	1.71(0.69)	0.0129
	AA	Yes	378	106.80±9.52	-0.34(0.67)	0.6110	70.07±7.37	1.83(0.54)	0.0007
	GA	Yes	362	107.72±9.42	0.41(0.68)	0.5481	69.98±7.99	1.66(0.55)	0.0024
	GG	Yes	111	107.62±8.84	0.56(1.00)	0.5730	70.10±7.08	1.39(0.81)	0.0859
	P 交互项					0.9384			0.0408
rs2298850	GG	No	564	107.93±9.87	Ref		69.27±7.97	Ref	
	CG	No	437	107.46±9.59	-0.39(0.59)	0.5120	68.57±8.31	-0.67(0.49)	0.1729

	CC	No	99	107.32±10.4	-0.73(1.01)	0.4716	68.16±8.31	-1.26(0.84)	0.1324
	GG	Yes	339	107.64±9.13	-0.20(0.64)	0.7597	69.85±7.72	0.43(0.53)	0.4137
	CG	Yes	385	106.73±9.28	-1.17(0.62)	0.0586	69.99±7.36	0.54(0.51)	0.2942
	CC	Yes	122	107.84±10.4	-0.21(0.93)	0.8193	70.89±7.83	1.55(0.77)	0.0434
	P 交互项					0.8360			0.0361
rs3755967	CC	No	575	107.95±9.81	Ref		69.26±8.03	Ref	
	CT	No	438	107.39±9.62	-0.50(0.59)	0.3894	68.49±8.25	-0.75(0.49)	0.1234
	TT	No	98	107.40±10.4	-0.70(1.01)	0.4892	68.14±8.35	-1.27(0.84)	0.1297
	CC	Yes	345	107.65±9.17	-0.23(0.63)	0.7132	69.70±7.80	0.29(0.53)	0.5802
	CT	Yes	383	106.86±9.28	-1.12(0.61)	0.0697	70.07±7.34	0.61(0.51)	0.2318
	TT	Yes	123	107.81±10.3	-0.30(0.92)	0.7468	70.88±7.80	1.55(0.77)	0.0426
	P 交互项					0.7820			0.0173
rs4588	GG	No	571	107.91±9.83	Ref		69.20±8.02	Ref	
	GT	No	439	107.42±9.60	-0.43(0.59)	0.4582	68.61±8.32	-0.60(0.49)	0.2202
	TT	No	98	107.40±10.4	-0.68(1.01)	0.5019	68.04±8.26	-1.35(0.84)	0.1098
	GG	Yes	341	107.67±9.14	-0.19(0.64)	0.7656	69.78±7.79	0.41(0.53)	0.4380
	GT	Yes	382	106.82±9.30	-1.09(0.62)	0.0770	70.02±7.34	0.63(0.51)	0.2221

	TT	Yes	124	107.75±10.3	-0.32(0.92)	0.7262	70.79±7.83	1.52(0.76)	0.0472
	P 交互项					0.8569			0.0323
VDR									
rs2228570	GG	No	40	104.25±7.70	Ref		71.75±6.99	Ref	
	GA	No	74	107.00±8.37	2.73(1.50)	0.0702	72.23±6.79	0.45(1.23)	0.7158
	AA	No	23	105.04±6.09	1.56(2.01)	0.4384	71.39±6.59	0.32(1.64)	0.8455
	GG	Yes	48	109.67±8.31	5.95(1.65)	0.0003	72.88±5.07	0.99(1.34)	0.4627
	GA	Yes	106	105.53±7.32	1.64(1.45)	0.2568	72.05±6.06	-0.20(1.18)	0.8656
	AA	Yes	37	106.38±8.01	3.18(1.78)	0.0753	72.00±6.20	-0.30(1.45)	0.8391
	P 交互项					0.0434			0.4043
rs2238136	CC	No	99	105.86±8.19	Ref		72.58±6.68	Ref	
	TC	No	37	106.05±7.17	0.75(1.51)	0.6182	70.05±6.74	-2.27(1.19)	0.0576
	TT	No	1	—	—	—	—	—	—
	CC	Yes	126	106.75±7.92	1.51(1.12)	0.1764	71.95±5.70	-1.19(0.88)	0.1771
	TC	Yes	57	106.40±7.55	1.63(1.35)	0.2292	72.54±6.03	0.06(1.07)	0.9536
	TT	Yes	8	108.75±9.91	2.46(2.87)	0.3922	74.75±6.67	1.47(2.27)	0.5193
	P 交互项					0.8841			0.0348

Abbreviations: VitD, vitamin D; SBP, systolic blood pressure; DBP, diastolic blood pressure.

Adjusted for pre-pregnancy BMI, maternal age, gestational weight gain, educational level, parity, basal blood pressure and the seasons of blood pressure measurement.

Table S12. The association of single SNP and VitD at T3 with blood pressure at T3.

SNP	Genotypes	VitD deficiency	N	SBP, mmHg			DBP, mmHg		
				Mean ± SD	β(se)	*P*	Mean ± SD	β(se)	*P*
CYP24A1									
rs2248137	GG	No	339	109.30±10.6	Ref		70.07±8.04	Ref	
	GC	No	106	109.65±9.29	0.39(1.11)	0.7238	72.53±6.81	1.85(0.86)	0.0307
	CC	No	195	109.11±9.97	-0.11(0.88)	0.8986	70.01±8.54	-0.18(0.68)	0.7914
	GG	Yes	131	110.80±10.5	1.39(1.03)	0.1767	71.18±7.61	0.51(0.80)	0.5200
	GC	Yes	63	107.21±8.66	-2.33(1.39)	0.0945	73.19±6.15	1.94(1.08)	0.0736
	CC	Yes	90	107.82±9.86	-1.86(1.17)	0.1138	71.60±7.25	0.98(0.91)	0.2822
	P 交互项					0.0361			0.6400

Abbreviations: VitD, vitamin D; SBP, systolic blood pressure; DBP, diastolic blood pressure.

Adjusted for pre-pregnancy BMI, maternal age, gestational weight gain, educational level, parity, basal blood pressure and the seasons of blood pressure measurement.

Table S13. The association of single SNP and VitD at T2 with blood pressure at T3.

SNP	Genotypes	VitD deficiency	N	SBP, mmHg			DBP, mmHg		
				Mean ± SD	β(se)	*P*	Mean ± SD	β(se)	*P*
CYP24A1									
rs6127118	GG	No	220	109.82±9.86	Ref		71.15±7.78	Ref	
	AG	No	371	109.69±10.8	-0.42(0.85)	0.6243	69.98±7.79	-1.34(0.65)	0.0378
	AA	No	17	106.82±6.82	-3.29(2.52)	0.1920	71.12±6.00	-1.07(1.92)	0.5781
	GG	Yes	186	107.68±8.99	-1.87(1.00)	0.0616	71.19±7.85	-0.08(0.76)	0.9161
	AG	Yes	305	109.35±10.5	-0.64(0.89)	0.4700	72.02±7.97	0.58(0.67)	0.3931
	AA	Yes	24	107.67±10.1	-2.68(2.16)	0.2148	73.67±5.86	1.79(1.64)	0.2754
	P 交互项					0.2294			0.0236

Abbreviations: VitD, vitamin D; SBP, systolic blood pressure; DBP, diastolic blood pressure.

Adjusted for pre-pregnancy BMI, maternal age, gestational weight gain, educational level, parity, basal blood pressure and the seasons of blood pressure measurement.
